# Are Mushrooms Parametric?

**DOI:** 10.3390/biomimetics7020060

**Published:** 2022-05-10

**Authors:** Dilan Ozkan, Ruth Morrow, Meng Zhang, Martyn Dade-Robertson

**Affiliations:** 1 School of Architecture, Planning and Landscape, Newcastle University, Newcastle upon Tyne NE1 7RU, UK; ruth.morrow@newcastle.ac.uk (R.M.); martyn.dade-robertson@newcastle.ac.uk (M.D.-R.); 2 Applied Sciences, Northumbria University, Newcastle upon Tyne NE1 8ST, UK; meng.zhang@northumbria.ac.uk

**Keywords:** fungal fruiting bodies, parametric design thinking, plasticity, linearity, non-linearity

## Abstract

Designing with biological materials as a burgeoning approach in the architecture field requires the development of new design strategies and fabrication methods. In this paper, we question if designers can use a parametric design approach while working with living materials. The research uses fungi as a biomaterial probe to experiment with the parametric behavior of living systems. Running design experiments using fungi helps to understand the extent to which biological systems can be considered parametric and, if so, what kind of parametric systems they are. Answering these questions provides a method to work with complex biological systems and may lead to new approaches of fabricating materials by tuning the environmental parameters of biological growth.

## 1. Introduction

There is a growing interest in harnessing living systems in the fabrication of materials and structures. Biological systems are capable of self- assembling complex materials and composites in highly energy efficient ways. While we make use of the materials provided by nature after the organism that created them is dead, utilizing living process may offer new methods of material assembly. These methods, however, will also require novel design tools and a new understanding of the relationship between the designer and their materials.

A promising group of organisms for biological material fabrication is fungi. For example, fungi can be manipulated at various scales for different purposes, such as in leather form with similar texture to animal leather and as a binder for bulk material (as mycelium composites). It also can act as a functional material when it is still alive, to form networks for microorganisms (see Fiber Highways Project [1]), or as a sensor (see Fungal Architecture project [2]). Most of these projects utilize fungal mycelium; however, few design projects address the fruiting body. Unlike mycelium, the fruiting bodies of many fungus species exhibit complex morphologies and self-assemble without the ‘scaffold’ of a substrate or aggregate. While we tend to harvest the fruiting bodies as food, the morphological complexity and their sensitivity to environmental conditions, as well as their speed of growth make them especially suitable for studies on how biological systems fabricate complex forms and materials. To this end we provide an early study in which the fruiting bodies of a well-studied fungal species (*Pleurotus ostreatus*) are shown to be somewhat controllable given their sensitivity to key environmental parameters. They were used as a biomaterial probe to test the concept of biological parametrics [3].

Biomaterial probes are defined as experiments that are carried out on biological materials or fabrication strategies without designed goals, but which are used to understand the factors influencing a biological system [4]. As Ramirez-Figueroa explains, it focuses on design explorations which show how the practice of design is transformed and redefined by using living systems. Although mycelium was used as a material probe in the preliminary experiments, the main design experiments were conducted here using fruiting bodies [5]. The goal of the design experiments was to intervene in the fruiting body formation of oyster mushrooms by altering the environmental factors for growth.

### 1.1. Biological Parametrics

Parametric design is a broad concept that connects data to the design of form and structure. Often synonymous with generative design, the role of the designer in a parametric design process is not to design the form of the object or system directly but rather to define the key controlling parameters and their relationships [6]. In Architecture, parametric design is often associated with the development of complex organic forms derived from initial conditions created by, for example, site mappings or simulations of use and function [7,8].

There is an analogy between parametric and biological processes in that in many examples of biological growth, especially in plants and fungi, the form of the organism is often, in part, derived from an interaction with environmental factors, including, access to sunlight and nutrients, physical constraints and barriers and interaction with other organisms. To some extent we already intervene in these biological processes in agriculture. A tomato, cultivated in the highly controlled, nutrient rich environment of a greenhouse, for example, could be described as ‘parametrically designed’. Refined crafts such as bonsai tree growing are also examples of intervening in biological growth with specific forms in mind. The Bonsai tree is produced through direct and ‘coercive’ control through the ‘directing’ of branches and the severe limitations of nutrients to keep the trees in dwarf form. In design terms this cultivation approach is more akin to direct control than parametric design, which implies a separation between the intervention (through data) and the generated design outcome.

Our research into fruiting bodies (of *P. ostreatus* known as *oyster mushrooms*) has, however, suggested that, for certain biological systems, a parametric approach to their ‘design’ and cultivation may be possible. To this end the paper will introduce the concept of biological parametric design as a fabrication strategy through design experiments which investigate the relationship between environmental parameters and fungal fruiting body morphology.

### 1.2. Plasticity

While it is often stated that DNA is the ‘blueprint of life’, biological systems are only partially shaped by the information contained in genes. Biological systems are subject to epigenetic influences i.e., environmental conditions which will cause genes to activate or not [9]. This relationship between phenotype and environment is sometimes referred to as plasticity and can be measured in terms of the degree of variation between organisms given the same genome [10]. Plasticity is exhibited at different stages of an organism’s life. Here, however, we will focus on developmental plasticity of mushroom fruiting bodies which lead to a variation in morphogenesis and final form. The concept of plasticity implies a pliability of developmental processes which may, we suggest, enable human intervention in direct parametric control.

As Dade-Robertson discusses, these indirect methods of affecting a living material through environmental parameters use “nature’s own agencies” without human imposition through “forcible constraints” such as cutting and molding the organism, or genetic manipulation [10]. A question remains, however, as to what degree this plasticity is amenable to a parametric approach. Biological systems and processes often exhibit non-linear behavior with, for example small changes in environmental conditions creating tipping points and leading to developmental outcomes that are not easy to attribute to single or limited sets of parameters and/or where the same effect does not always cause the same results [11]. An organism growing under exactly the same environmental conditions can form different morphologies. It is the non-linear behavioral pattern of the living materials that leads to an abundance of variations in the final product. Biological systems are also subject to noise and exhibit cell-to-cell variation and emergence where outcomes are not easily reducible to the behavior of parts. This biological complexity, therefore, challenges a parametric approach, and at the same time requires designers to have deep knowledge about the biological materials and bioprocess for the fabrication of the materials. Designers need to explore the value ranges and tipping points where the organism presents a linear change, so (if applicable) they can apply parametric design principles.

### 1.3. Prior Work

There are a number of notable precedents for a parametric approach to fabricating with biological systems outside the context of fungus. The works of Jiwei Zhou et al., Thora Arnardottir et al., and Neri Oxman et al., using plant roots, bacteria, and silkworms, respectively, show approaches to influence the environmental conditions of living organisms to achieve a desired material [9,10,11]. For instance, Arnodottir uses urease producing bacteria to calcify sand, creating cemented columns of material, without including digital tools to control the parameters [12]. By altering the cast sizes, inlet positions for nutrients and reactants, she shows that parameters which affect biological growth can be influenced. The influence of the parameters can be predicted while creating cast materials and the final form of the cemented columns does not have to be dictated by the shape of the cast. More complex forms emerge because of the interaction of these biological and environmental factors. In the case of the Silkworm Pavilion-II project by Oxman et al., they guide silkworms to cover the woven surface of the pavilion [13]. The distribution of the silkworms was controlled by heat, gravity, and light as variables. Since environmental conditions were directly linked to silk production, they could spread the fibers homogenously as they intended. Zhou et al., uses plant roots to test digital biofabrication strategies for product design purposes [14]. They fabricate self-supported 3D structures by altering the growth media, direction of gravity and porosity of their digitally fabricated mold. These variables allowed them to manipulate plant roots, since the nutritional richness and the force of gravity have an impact on the root growth [14].

In each example above, designers initially define the environmental factors (in a parametric manner) as variables they can work with to manipulate the final outcome. In each case they have shown that, to some extent (within a value range) there is a somewhat predictable relationship between environmental parameters and specific material outcomes. The outcomes of these processes also exhibit variations, however, and this challenges notions of fabrication tolerances.

### 1.4. Focus

This paper extends these works on biological parametric design by reporting four design experiments using fungal fruiting bodies. In each case the objectives are to find the environmental parameters responsible for different fruiting body morphologies and to see whether such morphologies can be predicted. The fruiting bodies of the selected fungus have the benefit of being complex, in terms of morphology but also plastic, in that they exhibit significant phenotypic variation given the same genetic information. They can also be grown quickly. These experiments seek to answer the question: To what extent is mushroom growth parametric?

## 2. Materials

Oyster mushrooms (*P. ostreatus*) were used in this study because of their fast growth (compare to other species used in the design field such as *ganoderma resinaceum* and *trametes versicolor*) and the wide variety of known fruiting body morphologies due to their gastronomic use, indicating a high level of developmental plasticity [15]. In addition to the rapid growth rate and plasticity, fungal fruiting bodies possess totipotency. Totipotency describes the ability of a cell to divide and produce all the differentiated cells of an organism autonomously [16]. This means if even a tiny amount of mushroom tissue is transplanted onto a nutrient medium, it can initiate new growth [15]. Totipotency enables the harvested cells to be used as the basis for a new experiment.

**Figure 1:**
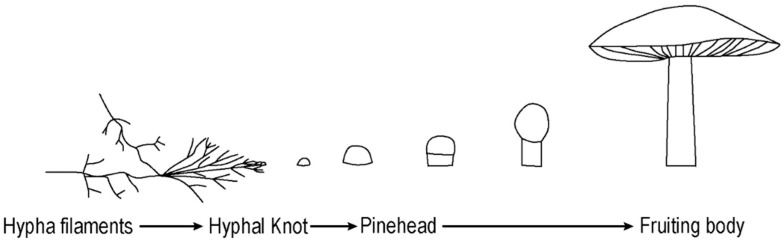
The developmental path of the fungal reproductive phase.

In each experiment the mushroom growth followed a common and well described developmental pathway starting with the vegetative phase (hypha growth), which continued to the reproductive phase (fruiting body formation) (Figure 1) [17]. Hypha filaments transform to the fertile tissue of a fruiting body under suitable conditions. The organization of hyphae significantly changes while creating fruiting bodies. Normally, the filaments show positive autotropism by growing in an upwards direction; however while forming a fruiting body structure, they start to grow inwards and show negative autotropism [18]. This is due to hyphae forming a three-dimensional compound complex by interlocking with other hyphae structures, instead of simply forming an unconstrained mesh. The initial development of the fruiting body begins with a hyphal knot, which can be triggered by a disturbance such as an injury, edge encounter or changes in nutrient levels, temperature, or light exposure [18]. In the formation of *Basidiomycota* fungi, hyphae form knots by reducing their level of chitin and the knots become mushrooms by expansion and inflation of pre-existing hyphae. Depending on the species’ phototropic requirements, the progress can proceed with the introduction of light that leads to cellular differentiation [17]. The formation of stipe (stalk), cap (pileus), and gill cells occur during this process, during which the mushroom takes on its characteristic appearance [15]. The spores are discharged from the surface of gills. Therefore, gills increase their surface area by folding, to allow the production of more spores.

## 3. Methods

### 3.1. Factors in the Morphogenesis of Mushrooms

In previous literature it has been shown that different mushroom species adapt to the environment they occupy to maintain their life and chances of reproducing [19]. Mushroom morphology is connected to the transportation of spores, where the fungi adopt forms that optimize the diffusing of spores [15]. For instance, the umbrella shape of mushrooms comes from the upward development of stipes under the influence of light, whereas the gills that diffuse the spores develop downward and are affected by gravity [19]. However, the umbrella shape can be changed by altering the direction of light and gravity [19].

The major factors that affect the form of mushrooms depend on the species. Bellettini et al. has conducted experiments which show the key parameters affecting the mushroom morphology of oyster mushrooms: air temperature, light, humidity, CO_2_ levels, gravity, substrate amount and size [20]. These factors influence the cap and stalk’s shape, size, and surface finish of oyster mushrooms [21]. Therefore, in this study humidity, CO_2_ level, gravitational force and substrate amount are used as variables to test the parametric qualities of mushrooms. Light duration and temperature are kept as constant values since we found across our interaction with fungi that they are more effective in initiating the mushroom formation rather than affecting mushroom morphology. High humidity environments provide favorable conditions for mushrooms to thrive in and bear fruit [18]. Different sources state that using 90–95% humidity or using 80–85% humidified culture room as well as spraying their fungi three times a day helps to achieve the optimal mushroom yield [20,22]. Stalk thickness tends to decrease with the decrease in the level of humidity, since there is not enough water for mushroom development [18,23].

A change in CO_2_ concentration also triggers different stages of the fungal life cycle and affects the morphology of mushrooms. During the development of mushrooms, respiration activity increases, so the preferred CO_2_ level decreases. While the preferred CO_2_ concentration is 2000–2500 mg/L for mycelium growth, it decreases to 1500–2000 mg/L for fruiting body development. If the CO_2_ level remains high, the cap formation may not occur [20]. High CO_2_ concentration blocks pileus formation while boosting stalk elongation because the cell wall is affected by elevated CO_2_ levels [19].

Many mushroom stalks possess negative gravitropism [24] as the fruiting bodies grow in the opposite direction of gravity and bending of the stalk occurs at the upper region closest to the cap [19]. In the literature, the substrate mass has often been studied as it affects the size and number of mushroom blooming because of the impact on nutrient availability.

### 3.2. The Experimental Design

From the literature above the effects of humidity, CO_2_ levels, gravitational force and substrate amount were chosen as variables as these had the potential to have the most significant impact on mushroom morphology. To validate this decision, a series of experiments were conducted testing the effect of different conditions in isolation. The experiments were carried out during the COVID-19 period and hence some of the experimental setups were improvised around the available equipment and facilities.

Humidity and CO_2_ levels in the experiments were controlled by a growth chamber that consists of an Arduino UNO (connected to a laptop), Arduino sensors (DHT11 air humidity and temperature sensor, SEN0219 infrared CO_2_ sensor, V1.0 soil moisture sensor and HC-SP04 ultrasonic distance sensor) and devices (12V DC fan, humidifier, 450 nm LED blue light source and 75 watt heat bulb) [3]. The chamber also helped to keep temperature and light exposure stable. Only one variable was changed at a time and the others were kept constant for each experiment (Table 1).

Each set started with the same substrate ratios with 25% of strawbale, 25% of wood shavings, and 25% of coffee grounds. Straw was blended in a Nutri Ninja Blender & Smoothie Maker 900 W for 5 s to a homogeneous mixture. The wood shavings and coffee grounds were not blended since they already had uniform size. The substrates were prepared and sterilized in an autoclave at 121 °C for 15 min. This mixture was then seeded with 25% of oyster mushroom spawn (*P. ostreatus*) from GroCycle-UK, and sealed in (10 × 10 × 3 cm) plastic boxes, in the dark, at ambient temperature. The experiments ran for 29 days. After an initial three weeks of growth the samples were exposed to different environmental conditions for eight days in the growth chambers. All experiments were conducted in triplicate.

By altering the parameters incrementally across different experiments, as seen in Table 1, we were able to measure the scale effect of different environmental conditions and relate specific parameters with mushroom dimensions.

#### 3.2.1. The Humidity Experiment

The variable of humidity level was set to four different levels as discussed in our previous paper and seen in Table 1 [3].

#### 3.2.2. The CO_2_ Experiment

The variable of CO_2_ level was set to three different levels as explained in our previous paper and seen in Table 1 [3].

#### 3.2.3. The Gravity Experiment

**Figure 2:**
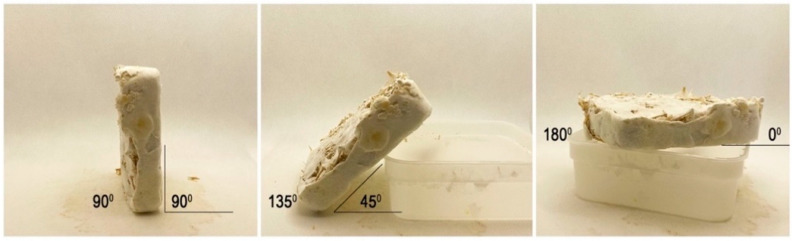
The positioning of the mushrooms in the gravity experiment.

In this experiment the angle of growth was tested. The effect of gravity upon the growing mushroom was adjusted as a means of support by using the aforementioned plastic containers. After being removed from the containers, the mycelium tiles were kept in 90°, 135° and 180° angles, as seen in Figure 2. The samples with 180° angles were positioned on a box. Lifting them prevented moistening and mushroom growth on the contact surface.

The experiment was repeated under 2000 ppm CO_2_ level. In this way, it was possible to see the effect of gravity on caps in different sizes.

#### 3.2.4. The Substrate Amount Experiment

**Figure 3:**
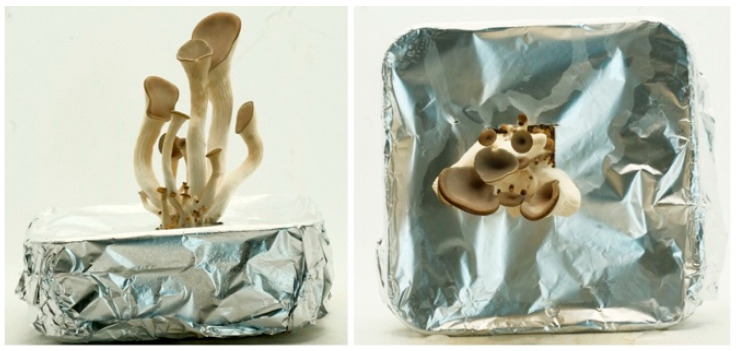
The preparation of the samples for the substrate amount experiment.

In this set of experiments, the effect of substrate amount on mushroom size was tested. 40 g, 80 g, 120 g and 160 g mycelium and various substrates were mixed in the ratio of 25% of strawbale, 25% of wood shavings, 25% of coffee grounds, and 25% of mushroom spawn, as mentioned before. All mixtures were kept in (10 × 10 × 3 cm) plastic box and covered with aluminum foil with a 4 × 4 cm hole in the middle of one of the widest surfaces, as seen in Figure 3. The aim of guiding the mushroom growth from a single opening was to limit the number of fruiting bodies, thus, to prevent overcrowding, to focus on the size of the mushrooms.

### 3.3. Measuring the Results

The mushroom morphology was documented at the end of day 27, through photography (Fujifilm X-T2 with 80 mm lens), microscopy (Dino-Lite digital microscope at 70× magnification) and 3D scanning (EinScan-SE desktop scanner). These tools helped to analyze the overall mushroom forms by allowing for the digital measurement of dimensions of the caps and stalks [3].

**Figure 4:**
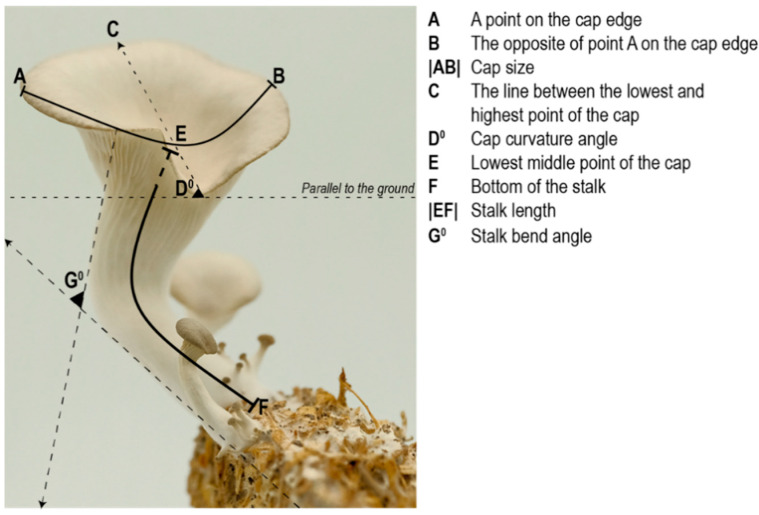
The locations of the measurement points.

The biggest mushroom from each replicate was selected as the most mature specimen (Figure 4). Measurements were made digitally using Rhinoceros 3D due to the difficulty in measuring delicate mushrooms of a small size.

The location of the measurement points for each specimen were standardized as follows:

- Capsize and stalk length are measured using curved lines. To measure the capsize |AB|, point-A is selected arbitrary on the cap edge, and point-B is located on the opposite side of the edge/point-A. To measure the stalk length |EF|, point-F is selected as the bottom of the stalk and point-E is selected as the lowest mid-point of the cap.
- The angle of the cap curvature (D°) is measured by:
  1. Drawing a line between the lowest and highest point on the cap edge.
  2. Measuring the angle between this line and the x-axis (parallel to the ground).
- The stalk curvature angle (G°) is measured by drawing two lines parallel to the stalk (one from underneath the cap, the other from the base of the stalk) and measuring the angle between these two lines.

## 4. Results

### 4.1. The Results of the Humidity Experiment

**Figure 5:**
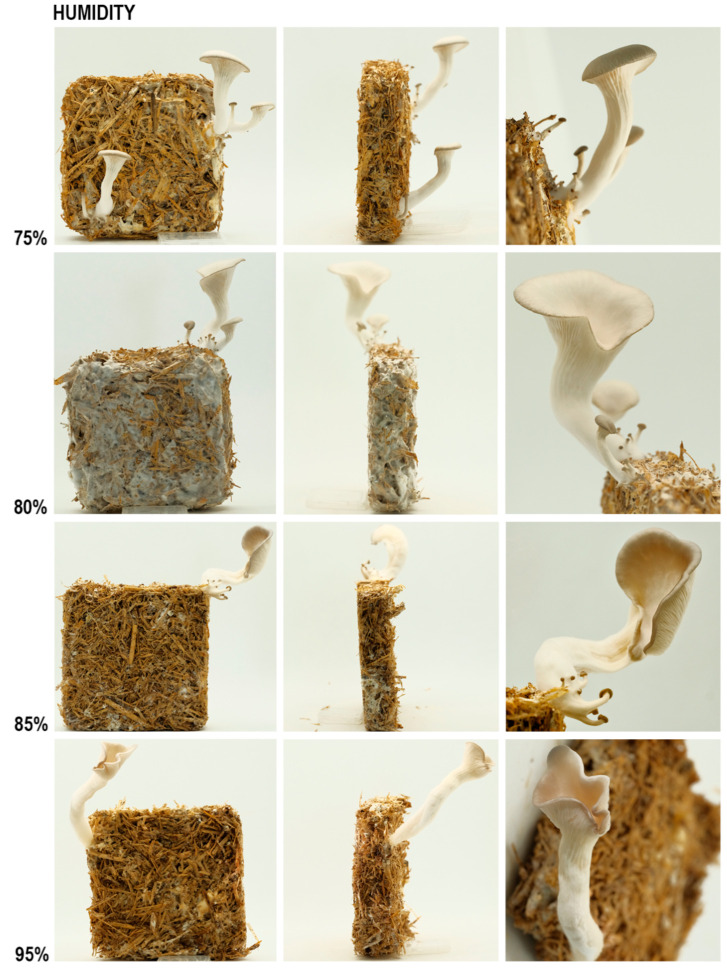
The results of the humidity experiment, front (**column 1**), side (**column 2**) and detailed (**column 3**).

In line with the previous study, humidity influences the curvature of cap edges and the stalks [3]. The replicates grown in the in-between conditions exhibit in-between morphologies. As we know from the humidity experiment, cap edge and stalk curvature increase with the increase in humidity level, as seen in Figure 5 and Table 2.

**Figure 6:**
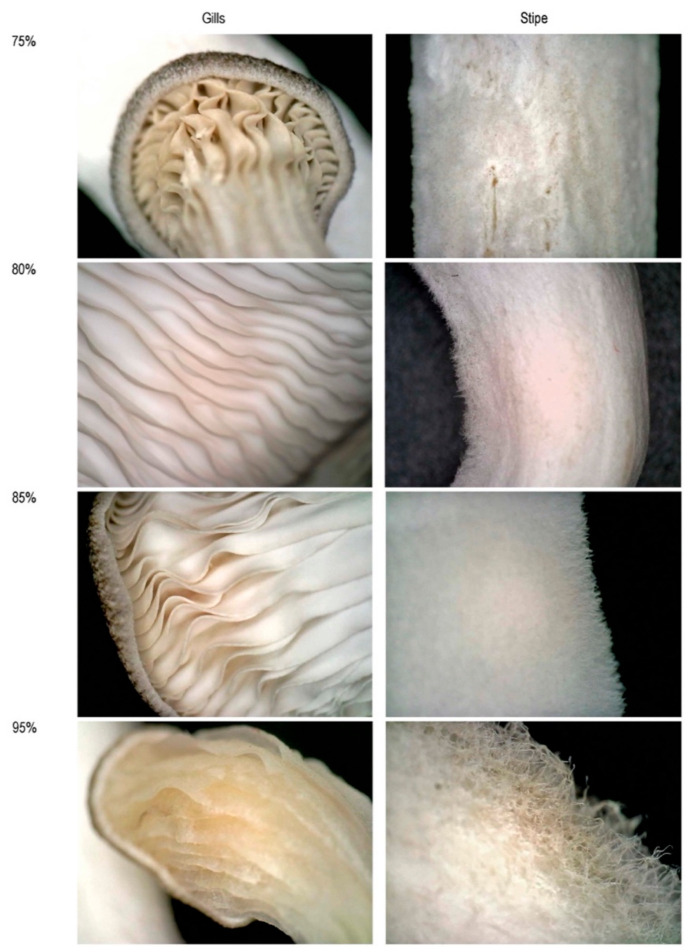
The comparison of gills and stipe under different humilities; images are captured using a Dino-Lite digital microscope at 70× magnification.

However, the cap sizes seem smaller in 80% humidity than the mushrooms grown in 75% humidity. This could be because the two outlier mushrooms grew bigger than expected and raised the average value, although this hypothesis needs to be validated by a bigger sample size. The texture of stalks and the depth of gills are qualitative results, and it can be observed from Figure 6 that in 80% humidity, gills are shallower, and stipes are hairier than 75%.

### 4.2. The Results of the CO_2_ Experiment

**Figure 7:**
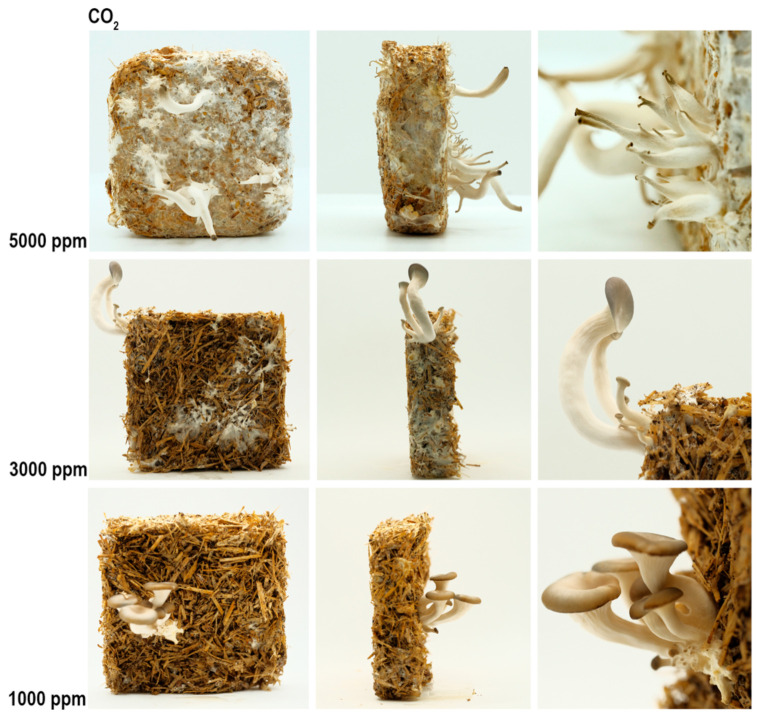
The results of the CO_2_ experiment, front (**column 1**), side (**column 2**), and detailed photos (**column 3**).

Altering CO_2_ levels has a significant effect on the cap size [3]. High CO_2_ decreases the cap size and inhibits mushroom maturation. As seen in Figure 7, although there are many sprouts, they elongate without cap formation. Their stalks get longer up to a certain level, as seen in Table 3. However, after a certain level (somewhere between 5000 ppm and 3000 ppm) the stalk length starts to decrease due to the high CO_2_ level.

### 4.3. The Results of the Gravity Experiment

**Figure 8:**
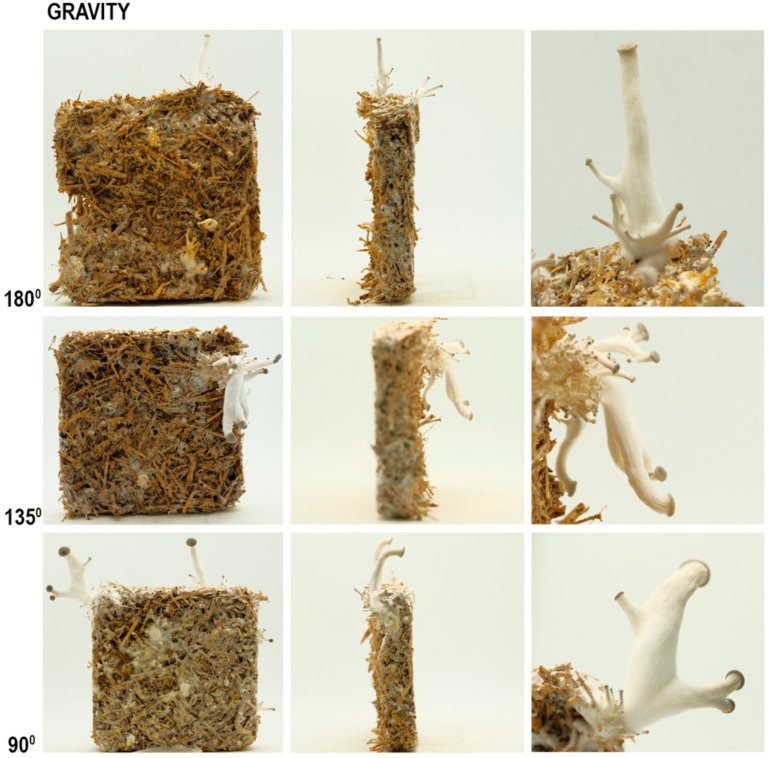
The results of gravity experiment at high CO_2_ (5000 ppm), front (**column 1**), side (**column 2**), and detailed (**column 3**) photos.

**Figure 9:**
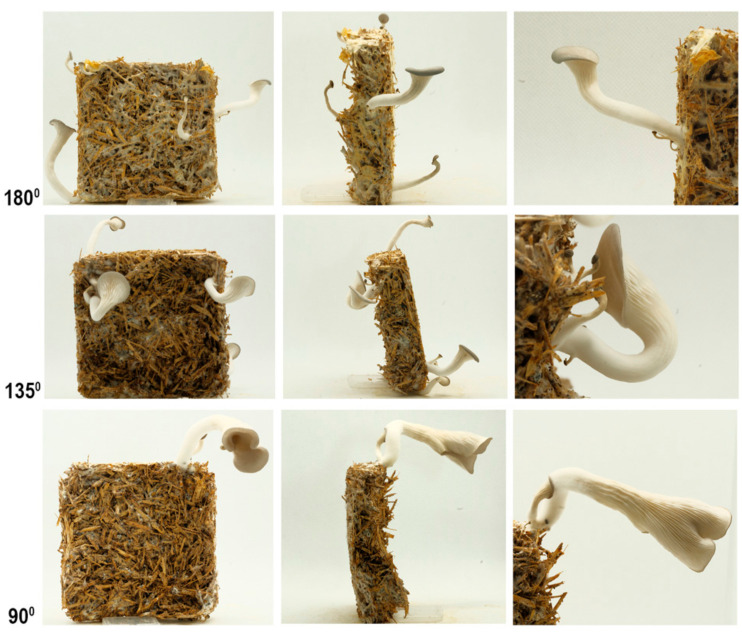
The results of gravity experiment at low CO_2_ (200 ppm), front (**column 1**), side (**column 2**), and detailed (**column 3**) photos.

As seen in Figure 8, there was a tendency for the fruiting body to grow vertically so the mushroom caps tended towards being parallel with the horizontal plane (Table 4). This led to the stalks being bent from underneath the cap, as they grow away from the tilted plane of the tile towards a vertical direction. As seen in Figure 9, the mushrooms grown at 5000 (high) and 2000 (low) ppm of CO_2_ presented the same behavior in terms of orientation.

In summary, gravity affects the orientation of the caps, which leads the stalks to curve accordingly but there is no significant impact on the size of the mushrooms.

### 4.4. The Results of the Substrate Amount Experiment

**Figure 10:**
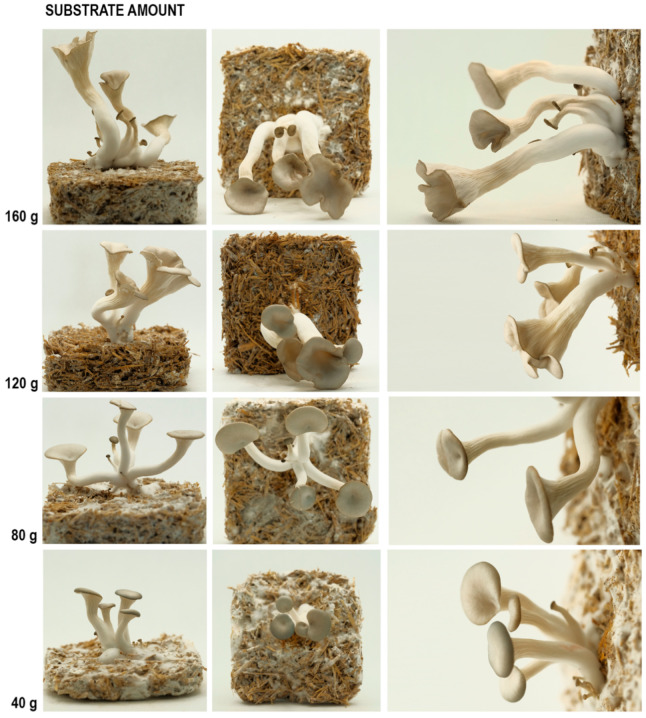
The results of the substrate amount experiment, side (**column 1**), top (**column 2**), and detailed (**column 3**) photos.

When the mushroom sizes and the number of sprouts is compared, as seen in Figure 10, an increase in cap size, stalk length and sprout number can be observed as the substrate amount increases (see Table 5). The reason for mushroom stalks growing with different curvatures is that they curled as they came out of the hole in the foil wrap.

The variable that has the most effect on the overall size of the mushrooms is the amount of substrates. Although an increase in humidity enlarges them to some extent, the substrate amount is the main determinant. Without sufficient substrates, the mushrooms cannot reach their maturity.

The samples grown in-between conditions exhibit in-between morphologies. As we know from the previous substrate amount experiment, the size of cap edges and the stalks get bigger as the substrate amount increases. All the curvature measurements in the 120 g mixture are somewhere between the 80 g and 160 g substrate amount. Although the sprout number is similar to the 80 g sample, it is less than the 160 g sample.

## 5. Discussion

**Figure 11:**
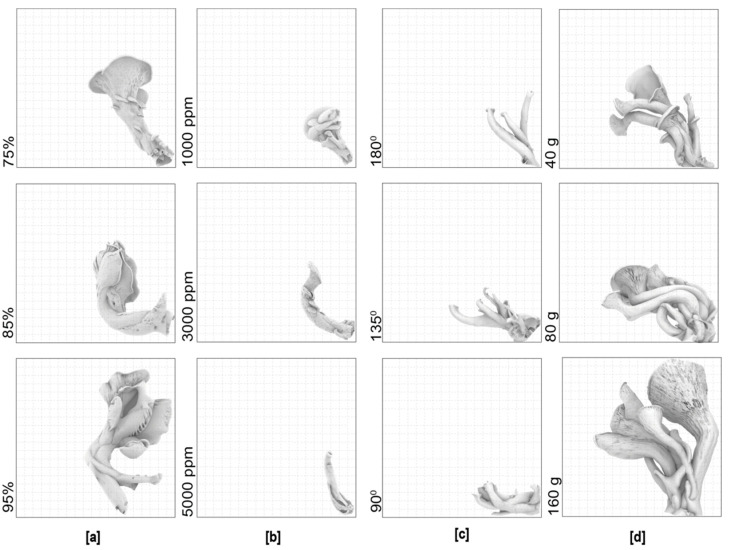
The overlay of the triplicate 3D scans of the mushrooms (at the same scale) grown under the same environmental conditions: The average sizes and the overlayed mushrooms in the (**a**) humidity (**b**) CO_2_; (**c**) gravity; (**d**) substrate amount experiment.

The experiments showed that the effect of different variables is often connected to similar affects in terms of mushroom morphology. For example, high humidity and substrate amount will affect mushroom size. And single variables are related to more than one affect. For example, humidity also affects curvature. Despite this, there is a fairly predictable relationship between each of the environmental parameters described in the experiments. Cap curvature is related to humidity, cap size is related to CO_2_, stalk bend is related to gravity, and overall mushroom size is related to substrate amount, as seen in Figure 11.

**Figure 12:**
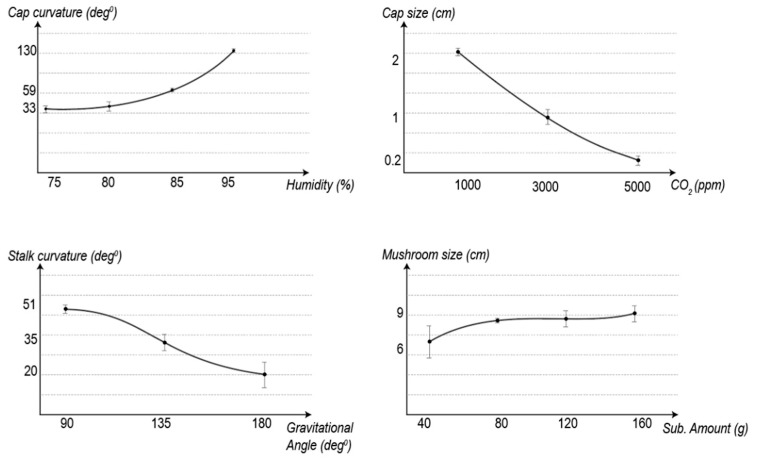
The mushroom growth—variables relationship.

The experiments also demonstrate that fungi exhibit linear parametric properties when a single parameter is changed, at least for the limited parameters and a single family of mushrooms tested here. As shown in Figure 12, each parameter change exhibits a distinct trend, although the high degree of variability exhibited in relation to mushroom size and substrate amount should be noted. More replicates are required in the future to achieve a higher significance of results. In all experiments, further replicates would need to be conducted to provide more significant relationships. Nevertheless, using these parameters, it should be possible to predict the morphology of mushrooms given specific parameters—within the range of values tested by the experiments.

Future research should also explore (1) the critical thresholds where growth is inhibited; (2) tipping points which lead the developmental pathway for the mushrooms to change; or (3) where normal development is critically disrupted where changing the variable no longer affects (or affects as expected) the mushroom morphology. For instance, mushroom growth may not be expected at very low humidity, or mushrooms cannot grow above a certain size even if the amount of nutrients are increased.

## 6. Conclusions

This paper asked the question: Is the growth of the mushroom fruiting bodies parametric? Or, more precisely: can mushroom morphology be predicted by altering the environmental parameters? As a designer, can we design fungal morphology using a parametric design approach? While we tend to think of biological systems as highly complex and non-linear systems, these albeit limited set of experiments have shown that given defined environmental conditions including factors such as CO_2_, humidity, orientation etc. we can see, within the limits of these experiments, linear relationships between environmental parameters and morphology outcomes. This points to the possibility of computational simulations for these systems and for the development of parametric-like software to estimate aspects of biological growth. It is also worth noting, however, that given the small sample size of the experiments (restricted to a single family of edible mushrooms) and the often significant, variation between mushrooms, we also need to recognize that these environmental factors are linked, and that mushroom morphology is highly sensitive to slight variations in conditions. This means that any attempt to model and predict the outcomes of different growth conditions will need to be, to some extent, probabilistic. The next step in this research will be to build such a model.

While the ability to alter the morphology of mushroom growth may be useful in, for example, agricultural contexts, these experiments are practical thought experiments. By trying to take a parametric design concept (which is well discussed in generative and computational design in architecture) and applying it to biological systems, we are revealing both its strength and weakness as a concept. With rapidly growing (literally and metaphorically) interest in the use of biomaterials in design and ideas of harnessing biological fabrication emerging from fields such as engineering living materials, this paper offered an alternative approach to the often gene-centric idea of engineering living organisms. We have shown that the developmental plasticity of mushrooms allows us access to control parameters outside the living cell of the mushroom that in turn remotely influence, rather than control, the material outcomes of mushroom growth. What is true for mushrooms may also be true of other sorts of biological systems.

Future work will need to extend the parameters explored and examine more closely their interrelationships as well as to increase the types of biological systems and processes amenable to change. It will also need to address the challenges of uncertainty of outcomes which are inherent in the design with biological systems.

## Figures and Tables

**Table 1:** The variables used for four experiments.

		Experiments													
		Humidity				CO_2_			Gravity			Sub. Amount			
Variables	Humidity (%)	95	85	80	75	80			75			80			
	CO_2_ (ppm)	2000				1000	3000	5000	5000			300			
	Sub. Amount (g)	55				55			55			40	80	120	160
	Gravity (degree)	90°				90°			90°	135°	180°	90°			
	Light (nm)	4 h, 450 nm				4 h, 450 nm			4 h, 450 nm			4 h, 450 nm			
	Temp. (°C)	20–22 °C				20–22 °C			20–22 °C			20–22 °C			

**Table 2:** The measurements of the humidity experiment.

		95%			85%			80%			75%		
		Rep-1A	Rep-2A	Rep-3A	Rep- 1B	Rep-2B	Rep-3B	Rep-1C	Rep-1C	Rep-3C	Rep-1D	Rep-1D	Rep-3D
Cap	Size (cm)	2.8	3.1	3.1	1.8	3.2	3.4	2.1	2.3	2.4	3.9	1.6	3.6
	Average size	3 ± 0.1			2.8 *±* 0.6			2.3 ± 0.1			3 ± 0.9		
	Curvature (degree)	120	127	145	53	64	60	30	38	31	31	32	36
	Average curv.	130.7 ± 7.4			59 ± 3.2			33 ± 3.3			33 ± 1.5		
Stalk	Length (cm)	5.6	5	4	4	4.9	4.4	3.5	4.1	4.7	4.7	4	3.2
	Average length	4.9 ± 0.5			4.4 ± 0.3			4.1 ± 0.3			4 ± 0.4		
	Curvature (degree)	104	133	140	85	83	90	51	78	81	54	61	76
	Average curv.	125.7 ± 2.9			86 ± 2.9			70 ± 7.8			63.7 ± 6.1		
Averge sprout number		2			4.3			4			3.3		

**Table 3:** The measurements of the CO_2_ experiment.

		5000 ppm			3000 ppm			1000 ppm		
		Rep-1A	Rep-2A	Rep-3A	Rep-1B	Rep-2B	Rep-3B	Rep-1C	Rep-2C	Rep-3C
Cap	Size (cm)	0.4	0.2	0.1	1.3	0.8	1	2.4	2.3	2.5
	Average size	0.2 ± 0.1			1 ± 0.2			2.4 ± 0.1		
	Curvature(degree)	31	2	5	41	53	40	8	7	9
	Average curv.	12.7 ± 11.8			44.7 ± 5.3			8 ± 0.8		
Stalk	Length (cm)	3.8	0.2	3	4.5	3.8	4.4	2	1.8	2.1
	Average length	2.3 ± 1.5			4.2 ± 0.3			2 ± 0.1		
	Curvature(degree)	66	79	73	44	41	39	53	61	55
	Average curv.	72.7 ± 5.3			41 ± 1.2			56.3 ± 3.3		
Averge sprout number		8.3			6			6.6		

**Table 4:** The measurements of the gravity experiment in high CO_2_.

		90°			135°			180°		
		Rep-1A	Rep-2A	Rep-3A	Rep-1B	Rep-2B	Rep-3B	Rep-1C	Rep-2C	Rep-3C
Cap	Curvature(degree)	6	12	0	61	35	16	44	19	35
	Average curv.	6 ± 2.4			37.3 ± 10.6			32.7 ± 10.2		
Stalk	Curvature(degree)	44	51	57	35	23	46	23	36	0
	Average curv.	50.7 ± 3.7			34.7 ± 6.6			19.7 ± 10.5		

**Table 5:** The measurements of the substrate amount.

		40 g			80 g			120 g			160 g		
		Rep-1A	Rep-2A	Rep-3A	Rep-1B	Rep-2B	Rep-3B	Rep-1C	Rep-1C	Rep-3C	Rep-1D	Rep-1D	Rep-3D
Cap	Size (cm)	1.3	1.2	2.9	2.2	2.3	1.8	3.4	3.7	1.5	3.9	2.5	2.8
	Average size	1.8 ± 2.1			2.1 ± 0.6			2.9 ± 2.7			3.1 ± 0.4		
Stalk	Length (cm)	5.3	2.9	5.1	5.8	6	5.2	5.8	6.7	5.2	6.7	5.4	6.3
	Average length	4.4 ± 1			5.7 ± 0.1			5.9 ± 0.4			6.1 ± 0.5		
Average sprout number		4			6			5			9		

## Data Availability

Not applicable.
